# Interpersonal and targeted rejection life stressors are proximal risk factors for suicidal ideation and behavior

**DOI:** 10.1017/S0033291725101414

**Published:** 2025-09-12

**Authors:** Lori N. Scott, Iulia Banica Malcolm, Sarah L. Brown, Evelyn M. Hernandez Valencia, Robert T. Krafty, George M. Slavich

**Affiliations:** 1 Department of Psychiatry, School of Medicine, University of Pittsburgh, Pittsburgh, PA, USA; 2 Ontario Shores Centre for Mental Health Sciences, Whitby, ON, Canada; 3 Department of Psychology, Florida State University, Tallahassee, FL, USA; 4 Department of Psychology, University of Rhode Island, Kingston, RI, USA; 5 Department of Biostatistics and Bioinformatics, Emory University, Atlanta, GA, USA; 6 Department of Psychiatry and Biobehavioral Sciences, University of California Los Angeles, Los Angeles, CA, USA

**Keywords:** life events, interpersonal stress, suicidal ideation, suicidal behavior, targeted rejection

## Abstract

**Background.:**

Although life stressors are known risk factors for suicide, the specific stressor types that most strongly precipitate suicidal outcomes, and on what timescale, remain poorly understood. Based on existing theory, we investigated whether objectively rated interpersonal stressors, especially social and targeted rejection stressors, are proximally associated with increased likelihood of suicidal ideation and behavior.

**Method.:**

Using an objectively rated contextual threat interview to assess stressful life events, and a timeline followback procedure for assessing suicide-related outcomes, we examined how the severity of four types of acute life events (i.e. non-interpersonal, interpersonal without social rejection, social rejection without targeted rejection, and targeted rejection) were temporally associated with the likelihood of same-day and next-day suicidal ideation and behavior over 16 months in 143 young adults (*M*_age_ = 25.27, *SD* = 4.65) with recent suicidal ideation or behavior.

**Results.:**

After controlling for prior-day suicidal ideation and non-interpersonal stressors, daily within-person increases in interpersonal stressor severity were related to higher odds of same-day (but not next-day) suicidal ideation. Additionally, greater increases in targeted rejection severity were uniquely related to increased likelihood of both same-day and next-day suicidal behavior after controlling for prior-day suicidal behavior and other types of stressors.

**Conclusions.:**

Interpersonal stressors are strong, proximal risk factors for suicidal ideation and behavior, and these effects are particularly strong for targeted rejection life events. Clinicians should thus assess recent interpersonal and, especially, targeted rejection stressors when evaluating acute suicide risk, and may reduce such risk by helping patients stabilize and strengthen their social relationships.

## Introduction

Suicide is the second leading cause of death in young adults in the United States (Centers for Disease Control and Prevention, 2024). Although prior studies have identified risk factors that tell us *who* is more vulnerable to suicide (Franklin et al., 2017), we still lack a clear understanding of which proximal social-environmental conditions predict acute, within-person increases in suicidal ideation and behavior (i.e. preparatory behaviors or attempts). The clarification of proximal risk factors for acute increases in suicide risk is critical to the development of more effective and efficient risk detection and intervention strategies to prevent suicide.

Several prominent theories emphasize stressful life events as risk factors for suicide (e.g. Klonsky, 2018; O’Connor, 2011; Van Orden et al., 2010). Consistent with these theories, research has demonstrated strong associations between experiencing life stress and increases in suicide-related outcomes (for reviews, see Liu & Miller, 2014 and Howarth et al., 2020). Interpersonal stress is theorized to play an especially important role in the generation of suicidal ideation (Glenn, Cha, Kleiman, & Nock, 2017; Van Orden et al., 2010; O’Connor, 2011). Indeed, research suggests that interpersonal stressors disrupt social relationships and increase negative beliefs such as perceived burdensomeness or thwarted belongingness, thereby increasing risk for suicidal ideation (Buitron et al., 2016; Glenn et al., 2022; Puzia, Kraines, Liu, & Kleiman, 2014).

Although several studies have demonstrated that negative interpersonal life events tend to precede increases in suicidal ideation and behavior (e.g. Bagge et al., 2023; Bagge, Glenn, & Lee, 2013; Bagge, Littlefield, Conner, Schumacher, & Lee, 2014; Conner et al., 2012; Husky et al., 2017; Nock, Prinstein, & Sterba, 2009), the types of interpersonal stressors that are most strongly predictive of acute increases in suicidal ideation and behavior remain unclear. Social rejection may be an especially potentinterpersonal stressor because it threatens to interrupt the maintenance of social bonds and stimulates a cascade of affective, cognitive, and biological changes that promote depression (Slavich et al., 2023) and suicidal ideation (Brown et al., 2019). Longitudinal evidence suggests that social rejection–related stressors, such as bullying, are prospectively associated with subsequent suicide-related outcomes (Cheek, Goldston, Erkanli, Massing-Schaffer, & Liu, 2020; Massing-Schaffer et al., 2019; Stewart et al., 2019, 2024; Winsper, Lereya, Zanarini, & Wolke, 2012). Evidence also points to subjective experiences of social rejection as a proximal risk factor for acute prospective increases in suicidal ideation in young adults (Victor, Scott, Stepp, & Goldstein, 2019).

A specific type of social rejection that may be especially pernicious is targeted rejection, characterized by the intentional rejection of the individual, isolated (i.e. exclusive) direct impact on the individual, and social demotion (i.e. loss of an existing relational tie or social status; Slavich, Thornton, Torres, Monroe, & Gotlib, 2009). Common examples of targeted rejection include being broken up with by a romantic partner and fired from a job. Massing-Schaffer et al. (2019) found that targeted rejection prospectively predicted suicidal ideation in adolescents over a subsequent 9-month follow-up period but was not associated with suicide attempts and no longer prospectively predicted suicidal ideation after controlling for depressive symptoms or baseline suicidal ideation. Another study found that adolescents with a recent suicide attempt had significantly more targeted rejection events in the previous 6 months as compared to ideators without attempts (Stewart, Pizzagalli, & Auerbach, 2024). Finally, some research has shown that targeted rejection is related to changes in inflammatory biology that have been implicated in depression and suicide (Murphy, Slavich, Chen, & Miller, 2015). To our knowledge, however, no research has examined whether targeted rejection is a proximal risk factor for subsequent acute increases in suicidal ideation or behavior over short time frames (e.g. daily). This is a significant gap considering that suicidal ideation fluctuates substantially over short periods of time (Kleiman & Nock, 2018) and evidence that suicidal ideation progresses toward suicidal behavior within 12 hours for a majority of individuals (Millner, Lee, & Nock, 2017). Given that targeted rejection can occur suddenly and without warning, it has the potential to generate rapid increases in suicidal ideation and mobilization to engage in suicidal behavior.

More broadly, despite the theoretical and clinical implications of understanding the impact of interpersonal stress exposure on acute changes in suicide risk, this literature has several limitations. First, prior studies of interpersonal stressors as proximal predictors of suicidal ideation or behavior have relied on participants’ self-reported stressor severity levels [e.g. using ecological momentary assessment (EMA)], which are subject to social desirability and mood-dependent recall biases (Monroe & Slavich, 2019). Although EMA is ideal for achieving a high degree of temporal granularity in the identification of proximal risk for intraindividual changes in suicidal ideation, it is not well-suited to examining suicidal behavior (which occurs at low base rates and is rarely observed in the limited timeframe of an EMA protocol) and does not allow investigators to distinguish between stressor exposure and response due to its reliance on self-report. In addition, studies that have examined objectively measured stressor exposure in relation to suicide-related outcomes have aggregated stress and suicide outcomes over long periods of time (e.g. months; for example, see Massing-Schaffer et al., 2019; Stewart et al., 2019, Stewart et al., 2024). Greater temporal precision is needed to understand the degree to which interpersonal stressor exposure operates as a proximal risk factor for acute increases in suicide risk and, indeed, mental health outcomes more broadly (Moriarity & Slavich, 2023).

Another limitation of prior research is that many studies have only examined stressor exposure as it relates to suicidal ideation, with far fewer studies examining suicidal behavior. Ideation-to-action theories of suicide risk (e.g. Klonsky & May, 2015; O’Connor, 2011; Van Orden et al., 2010) posit that different processes may increase risk for suicidal ideation and behavior, and that interpersonal risk factors are most important in the emergence of suicidal ideation. More studies examining both suicidal ideation and behavior as separate outcomes are needed to evaluate this theoretical assertion empirically. Moreover, little is known regarding which *types* of interpersonal stressors are more strongly and proximally associated with an increased likelihood of suicidal ideation and behavior. Although targeted rejection may be an especially potent predictor, few studies have examined targeted rejection specifically in relation to suicide-related outcomes, and no studies have done so with adults.

Targeted rejection may be a particularly important risk factor for suicidal outcomes in young adulthood, a developmental period that is characterized by major shifts in responsibilities and roles as well as the navigation of increasingly complex interpersonal relationships (Arnett, Žukauskienė, & Sugimura, 2014; Shulman & Connolly, 2013; Wood et al., 2018). With the increased emphasis during early adulthood on developmental tasks such as intimate relationship formation and career development, these changes bring new opportunities for targeted rejection experiences, with potentially higher stakes (e.g. relationship breakups, job terminations). A better understanding of how different types of stressor exposure are associated with near-term increases in suicidal ideation and behavior in at-risk young adults would have important implications for identifying acute risk and need for immediate intervention.

## Present study

To address the above-described limitations, we examined which specific types of objectively assessed life stressors are proximally related to retrospective daily reports of suicidal ideation and behavior using timeline followback interviews in an at-risk sample of young adults. To do so, we used a contextual threat approach to assessing life stressors that included both a comprehensive life stressor interview and an independent panel of trained raters who estimated the severity of each life event that participants experienced using extensive rules and guidelines (Brown & Harris, 1978). Based on the research summarized above, we hypothesized that greater within-person increases in severity of each type of interpersonal stressor (i.e. nonspecific interpersonal, non-targeted social rejection, and targeted rejection) would be uniquely associated with greater odds of suicidal ideation and behavior both concurrently (same-day) and prospectively (next-day) after controlling for non-interpersonal stress and prior-day suicidal ideation and behavior. We also explored whether rejection-related stressors were more strongly associated with suicidal ideation and behavior than non-rejection stressors.

## Method

### Participants

Participants were 143 young adult men and women between the ages of 18 and 35 (*M*_*age*_ = 25.27, *SD*_*age*_ = 4.65; 83.2% assigned female sex at birth) who reported suicidal ideation or behavior in the past 4 months and were receiving ongoing behavioral healthcare. See Table 1 for detailed sample demographic and clinical characteristics.

### Procedures

Participants were recruited from July 2019 through April 2023 for a longitudinal study of suicide risk. Participants completed assessments at baseline and at three follow-up visits (4-, 8-, and 12-months). When a follow-up visit was missed, information regarding the missing 4-month timeframe was collected at the next follow-up visit. The retention rate at the 12-month follow-up assessment was 85%. The life stress interviewers were postbaccalaureate, master’s-level, and doctoral-level clinicians who were trained by licensed doctoral-level clinical psychologists with oversight by the first author (L. Scott). Interview training was a multi-month process that included watching and scoring at least three recorded or live interviews, role-plays with study staff, and conducting at least three interviews with live supervision, post-interview feedback, and discussion of scores. All participants provided written and oral informed consent, and study procedures were conducted in accordance with the University of Pittsburgh’s Institutional Review Board (Protocol Number: STUDY18100158; initial protocol approval date 11/30/2018).

### Lifetime and current psychiatric disorders

For descriptive purposes, lifetime and current psychiatric disorders were assessed at baseline using the Structured Clinical Interview for DSM-5 Research Version (SCID-5-RV; First, Williams, Karg, & Spitzer, 2015) and selected items from the Structured Clinical Interview for DSM-IV Personality Disorders (SIDP-IV; Pfohl, Blum, & Zimmerman, 1997). Lifetime (at baseline) and past-4-month (at each assessment) suicidal ideation and behavior were assessed using the full Columbia Suicide Severity Rating Scale (C-SSRS; Posner et al., 2011), which assesses the graded level of suicidal ideation as well as distinct types of suicidal behavior (i.e. aborted, interrupted, and actual attempts, preparatory behaviors) during specific time frames.

### Episodic life stress

The UCLA Life Stress Interview (LSI; Hammen, 2019) was administered at each study visit to record episodic stressful life events at the daily level over the previous 4 months, thereby assessing life events over a total of up to 16 months (from 4 months pre-baseline through 1 year of follow-up). Training for administration of the LSI was conducted with consultation from the LSI author (C. Hammen), who provided training materials, direct instruction, and feedback on administration and scoring procedures. Episodic stress was defined as acute life events that occurred during the specific timeframe being assessed, had a distinct onset, and were distinguishable from chronic life difficulties.

To facilitate accurate recall, we presented a calendar and encouraged participants to use personally relevant dates (e.g. holidays, special events) and electronic records (e.g. text messages, e-mails). For each event, the interviewer collected a narrative description and asked follow-up questions to provide context (e.g. duration, desirability, impact on life domains, availability of environmental support or resources). Narratives were stripped of all subjective information, including suicide risk/events, psychopathology, and treatment history, and were then presented verbally to a team of two to four trained raters who remained blind to these participant characteristics that could have biased their stressor ratings. Raters asked clarifying questions as needed to understand contextual factors. Interviewers were trained to only respond to questions with objective information and not to reveal anything about the participants’ subjective experience or psychiatric disorders/symptoms in their answers. Each rater first gave their own independent rating of objective threat (i.e. how stressful or impactful the event would be to the typical person under identical circumstances/context, rated from 1 to 5 in half-point increments). Next, the team discussed discrepancies and arrived at a consensus objective threat rating. Events rated by consensus as having no or minimal impact (objective threat score = 1) were excluded from further rating and analysis.

The rating team then decided through consensus discussion whether each life event was interpersonal (i.e. involving interpersonal interactions, having clear and direct interpersonal consequences, or having a socially evaluative component) and, if so, whether it met the criteria for social rejection (i.e. the most salient feature of the event being rejection of the subject by another person[s]). Social rejection events were rated for whether they met the criteria for targeted rejection, which required: (1) Intent (clear intent to reject, such as a direct verbal statement ending or limiting the relationship, and not merely resulting from inaction, such as when people grow apart or lose touch); (2) Isolated impact (only the subject experienced the direct impact); and (3) Social demotion (loss of social status or severing of a relational tie). As with contextual threat ratings, raters could ask clarifying questions of interviewers to gather more objective information, and the team achieved consensus ratings through discussion. Initial training, supervision, and the materials used to score interpersonal and targeted rejection stressors were provided by G. Slavich, and the rating team was continuously supervised by L. Scott.

A total of 1,989 events were available for analysis, with 1,197 (60.2%) events rated as interpersonal, of which 263 were rated as social rejection and 99 were rated as targeted rejection. Most events (82.7%) lasted 1 day or less. In lieu of interrater reliability using independent rating teams, which was not feasible given the length of follow-up and the time-ntensive nature of consensus scoring procedures requiring multiple blind raters, we assessed interrater reliability of initial independent objective threat ratings from each individual rater provided prior to reaching consensus. The intraclass correlation coefficient (ICC) was calculated based on a mixed effects model with random effects, which accounts for the unequal number of raters per case. There was high agreement among raters for objective threat (ICC = .82). In addition, a randomly selected 10% of written life event narratives (prepared by interviewers and stripped of subjective information) were rated for interpersonal, social rejection, and targeted rejection categorization by an independent rater who was blind to participant clinical characteristics, subjective event information, and consensus team ratings. We calculated Fleiss’s kappa coefficients (κ) to assess agreement rather than Cohen’s kappa due to the fact that the independent raters were randomly selected, and not all cases were rated by the same raters. There was acceptable interrater agreement for interpersonal (Fleiss’ κ = .73), social rejection (κ = .71), and targeted rejection (κ = .61) ratings. Somewhat lower rater agreement for these ratings is not unexpected given that the reliability raters had to rely on written narratives only and did not benefit from the additional information shared during live team discussions with interviewers. Additional information about life event categorization is available in online - supplemental materials and at https://osf.io/wdk8y/files/osfstorage.

We computed scores for each individual at the daily level based on the sum of objective threat scores for events occurring on each day in each of the following categories: (1) Non-interpersonal; (2) Interpersonal without social rejection; (3) Social rejection without targeted rejection; and (4) Targeted rejection. For events that lasted multiple days, those event ratings were included in the calculation of stress severity scores on each applicable day.

### Suicidal ideation and behavior

Daily suicidal ideation and behavior were recorded over the past 4 months at each study visit using a timeline followback interview (Sobell & Sobell, 1992), which was administered after the LSI to avoid the influence of suicide-related events on reporting of LSI events. Information regarding the occurrence and type of any suicidal ideation or behavior during the timeframe was first obtained using the full C-SSRS (Posner et al., 2011) interview, with a calendar to specify dates of occurrence. Once it was established that a participant experienced any suicidal ideation or behavior during a specified 4-month timeframe with the C-SSRS, the calendar was used to identify the dates on which suicidal ideation and/or behaviors occurred. Participants reported any daily suicidal ideation (0 = *no*, 1 = *yes*), including either active or passive ideation (e.g. wishing to be dead or not wake up). Although information on specific types of suicidal behaviors (i.e. preparatory behaviors and aborted, interrupted, and actual attempts) was collected as part of the C-SSRS and TLFB procedures (see Table 2), base rates of each individual type of suicidal behavior were too infrequent to analyze separately. Therefore, in the analyses, daily suicidal behavior was operationalized as a binary outcome indicating the occurrence of any type of suicidal behavior that day (0 = *no*, 1 = *yes*). Daily number of standard alcoholic drinks and presence of non-suicidal self-injury and psychoactive druguse were alsorecorded. There were 64,731 days of timeline followback data, with an average of 422.66 (*SD* = 112.75) days per person. Suicidal ideation was reported during the timeline followback timeframe by all 143 (100%) participants and on 13,899 occasions, and suicidal behavior was reported by 45 (31.5%) participants and on 104 occasions. ICCs were .49 for suicidal ideation and .45 for suicidal behavior, indicating that a sizable proportion of variability was within persons.

### Data analysis

Preliminary descriptive analyses and bivariate correlations were conducted using SPSS 29. Hypotheses were tested using two-level logistic regression models with fixed effects using Bayesian Markov Chain Monte Carlo estimation with default (i.e. non-informative) priors in Mplus version 8.9 (Muthén & Muthén, 2023), in which daily observations (level 1) were nested within individuals (level 2). Both suicidal ideation and behavior were estimated simultaneously in each model as binary outcomes using a Bernoulli response distribution based on a probit link function. Bayesian estimation is advantageous for its superior performance, relative to alternative methods such as restricted maximum likelihood estimation, for multilevel models with binary outcomes (Asparouhov & Muthén, 2020). The use of non-informative priors allows the data to drive the estimation rather than introducing prior assumptions, which can lead to unintended bias when there is limited past research from which to draw informative priors (Asparouhov, Hamaker, & Muthén, 2018). Another benefit of Bayes estimation is the ability to decompose repeated measures into within- and between-persons variance using latent mean centering, which avoids well-demonstrated bias associated with observed mean centering (Asparouhov & Muthén, 2019). Because the variance is decomposed, within-person parameters can be interpreted as associations among within-person daily changes in constructs relative to one’s own average level, and between-person parameters can be interpreted as associations among between-person latent means over time. To further explore the influence of centering for our results, we examined each of the final models with no centering of life stressor variables, modeling the uncentered stressor severity variables only at the within-person level (as these scores would comprise both within- and between-person variance). The results of these models did not differ from those reported here, meaning that centering did not influence our findings.

We estimated one model for same-day effects and one for time-lagged effects (i.e. daily stress scores predicting next-day suicidal ideation and behavior), with each model examining non-interpersonal, interpersonal without social rejection, social rejection without targeted rejection, and targeted rejection stressor severity as time-varying predictors. We controlled for prior-day suicidal ideation and behavior in each model, which allows us to draw conclusions about within-person changes from day to day in these outcomes. We then conducted sensitivity analyses to determine whether results changed substantively when we added time-varying alcohol intake (number of standard alcoholic drinks), psychoactive drug use, and non-suicidal self-injury as time-varying covariates. Correlations among independent variables and among residuals of dependent variables were specified in each model. Differences in the magnitude of parameter estimates for the prediction of suicidal ideation and behavior by each type of stressor were examined using the ‘model constraint’ command. Adjusted odds ratios are presented as measures of effect size.

## Results

### Preliminary analyses

To identify potential between-persons covariates, we examined bivariate associations between person-level means of suicidal ideation and behavior (aggregated across timeline followback days) and participants’ age, sex, gender, sexual orientation, race, ethnicity, public assistance tied to low income, current major depressive disorder, number of days of timeline followback data, and number of life events. Female gender identification was associated with a lower incidence of suicidal behavior. Identification as gender diverse and receiving public assistance due to low income were both associated with a higher incidence of suicidal behavior. Having fewer days of timeline followback data was associated with a lower incidence of both suicidal ideation and behavior. All other bivariate correlations were nonsignificant. Hence, female gender, gender diversity, receipt of public assistance, and number of days of data were retained as between-person covariates in multilevel models. In tests for multicollinearity, the highest variance inflation factor value was 1.45, suggesting that multicollinearity was not problematic. Descriptive statistics and associations between person-level study variables are shown in Table 3.

### Primary analyses

**Figure 1.**
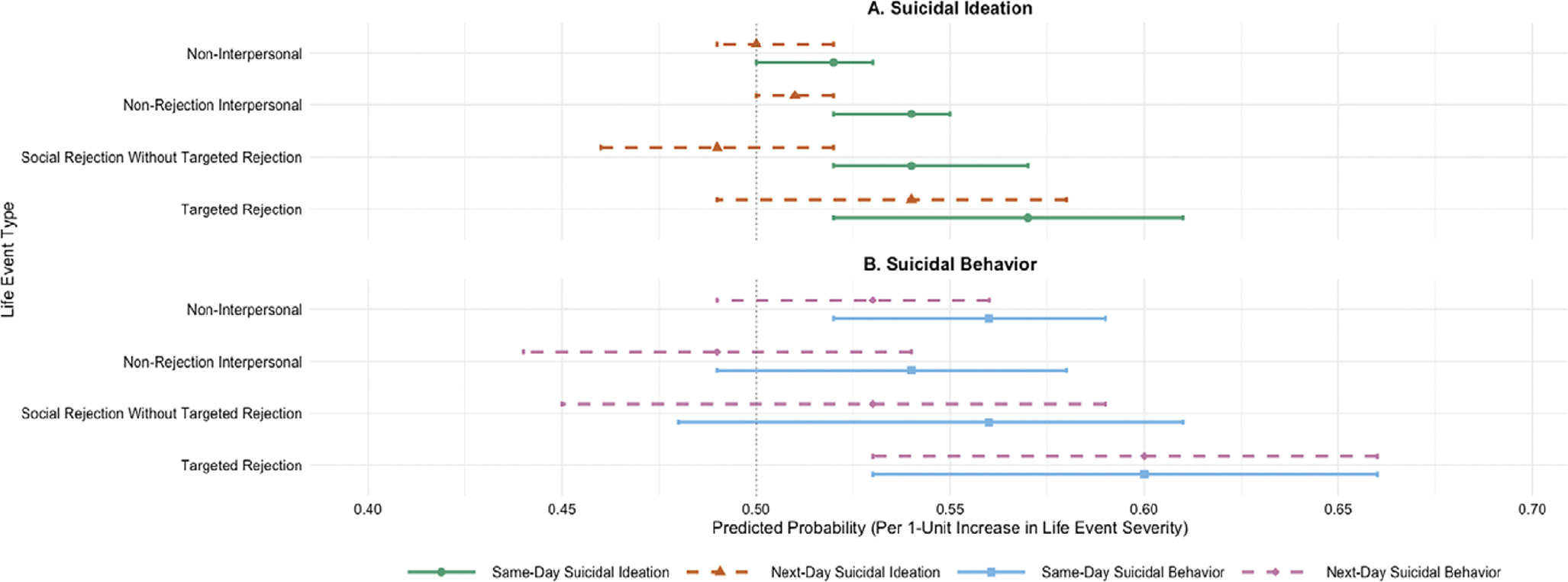
Predicted probabilities of suicidal ideation and behavior by life event type. *Note:* This figure illustrates predicted probabilities of suicidal ideation (panel A) and behavior (panel B) as a function of a one-unit increase in severity of each type of life event, separated by same-day (solid lines) and next-day (dashed lines) effects. Probabilities were calculated based on the model estimates reported in Table 4, with probability = odds/(1 +odds). Models controlled for previous-day suicidal ideation and behavior (not shown). Error bars represent 95% confidence intervals. Error bars that do not cross 0.50 indicate statistically significant effects (i.e. different from zero; *p* < .05). Probabilities above .50 can be multiplied by 100 for interpretation as the percent increase in chances of daily suicidal ideation or behavior with a daily one-standard-deviation increase (relative to a person’s average) in severity of each type of life event.

In both multilevel models, there were no significant associations between latent means of stressor severity scores and suicidal ideation or behavior on the between-persons level, and no significant differences in the magnitude of these estimates. The remainder of the results for stressor severity scores will therefore focus on the within-person effects (see Table 4 for all within-person statistics, and Figure 1 for predicted probabilities for within-person effects). The addition of time-varying covariates did not influence the results. The full model code and results, including models with time-varying covariates, are available at https://osf.io/wdk8y/files/osfstorage.

#### Same-day model.

As hypothesized, within-person daily increases (relative to a person’s mean levels) in all four types of objective stressor severity (i.e. non-interpersonal, interpersonal without social rejection, social rejection without targeted rejection, and targeted rejection) were significantly associated with same-day increases in the likelihood of suicidal ideation after controlling for prior-day suicidal ideation. In addition, partially consistent with hypotheses, within-person increases in non-interpersonal and targeted rejection stressor severity, but not other types of interpersonal stressors, were associated with an increased likelihood of same-day suicidal behavior after controlling for prior-day suicidal behavior. Difference tests revealed that non-rejection interpersonal stressors (B = 0.06, *SD* = 0.03, 95% CI = 0.01,0.11; *p* < .05) and targeted rejection stressors (B = 0.13, *SD* = 0.06, 95% CI = 0.01,0.26; *p* < .05) were more strongly associated with same-day suicidal ideation than non-interpersonal stressors. There were no other significant differences in the strength of model parameters.

#### Time-lagged (next-day) model.

Inconsistent with our hypotheses, none of the stressor severity scores prospectively predicted the likelihood of next-day suicidal ideation after controlling for prior-day suicidal ideation. Partially consistent with our hypotheses, only targeted rejection stressor severity was prospectively associated with an increased likelihood of next-day suicidal behavior after controlling for prior-day suicidal behavior, and the magnitude of this effect was significantly stronger than the effect of non-rejection interpersonal stressors (B = 0.27, *SD* = 0.10, 95% CI = 0.07,0.47; *p* < .05). There were no other significant differences between the strength of model parameters.

## Discussion

To address existing gaps in research linking life stressor exposure and suicidal outcomes, we examined how different types of objectively rated stressors related to the likelihood of same-day and next-day suicidal ideation and behavior, assessed retrospectively using timeline followback interview methods, in young adults. Results were largely consistent with our hypotheses regarding the importance of interpersonal stressors as proximal risk factors for both suicidal ideation and behavior, but revealed mostly concurrent rather than prospective associations, with the exception of targeted rejection stressors predicting next-day suicidal behavior, as further discussed below. For suicidal ideation, greater within-person increases in each of the three types of interpersonal stressors were associated with greater odds of same-day suicidal ideation, even after controlling for non-interpersonal stress severity and prior-day suicidal ideation. However, none of the stressor types significantly predicted next-day suicidal ideation after controlling for previous-day suicidal ideation.

One interpretation of these results is that the influence of life stressors on suicidal ideation is short-lived and does not extend to the next day. Given that we assessed life stressors and suicidal ideation at the daily level, we cannot rule out the possibility that life stressors may lead to even more short-term increases in suicidal ideation (e.g. imparting risk over the course of minutes to hours within days). We also do not know whether stressors occurred before or after suicidal ideation when both occurred on the same day; therefore, we cannot infer causality from same-day models. We can infer from our findings only that each type of interpersonal stressor is uniquely correlated with a within-person increase in the likelihood of suicidal ideation on the same day after controlling for the effect of prior-day suicidal ideation on current-day ideation. It is also possible that suicidal ideation may increase the likelihood and/or severity of experiencing subsequent stressors, particularly dependent interpersonal stressors, which would be consistent with a stress generation hypothesis (Liu & Spirito, 2019). Most likely, there are bidirectional associations among suicidal ideation/behavior and stress, which is also consistent with a stress generation model of symptom maintenance.

Based on our difference tests, both non-rejection interpersonal stressors and targeted rejection stressors were more strongly associated with same-day suicidal ideation than non-interpersonal stressors, but rejection-related stressors (including targeted rejection) were no more strongly associated with suicidal ideation than non-rejection interpersonal stressors. These results suggest that interpersonal stressors may generally be more strongly associated with near-term suicidal ideation than non-interpersonal stressors but call into question the idea that either social rejection or targeted rejection are more potent risk factors for suicidal ideation than other types of interpersonal stressors. Note, however, that even statistically significant differences in the magnitude of estimates were small, with predicted probabilities of same-day suicidal ideation associated with a one-unit increase in each type of stressor ranging from 52% for non-interpersonal stressors to 57% for targeted rejection (see Figure 1). These findings are consistent with suicide risk theories that emphasize broad-based interpersonal factors as important in risk for suicidal ideation (e.g. O’Connor, 2011; Van Orden et al., 2010) and further suggest that objectively assessed interpersonal stressors are proximal (i.e. near-term) risk factors for suicidal ideation that may be more impactful than non-interpersonal stressors.

Slightly different results emerged for suicidal behavior that point to targeted rejection as a unique proximal risk factor for suicidal behavior. Specifically, as hypothesized, targeted rejection was the only type of interpersonal stressor that was significantly associated with an increased likelihood of both same-day and next-day suicidal behavior after controlling for prior-day suicidal behavior and other types of stressor exposure. For every one-standard-deviation increase in targeted rejection severity on a given day, the chances of same-day and next-day suicidal behavior increased by 60% (see Figure 1). In addition, in our exploratory difference tests, the effect of targeted rejection stressors on next-day suicidal behavior was stronger than the effect of non-rejection interpersonal stressors on next-day suicidal behavior, but did not differ significantly in magnitude from the effect of any other type of stressor (i.e. non-interpersonal or social rejection without targeted rejection). In other words, targeted rejection appears to uniquely impact risk for suicidal behaviors compared to broad-based non-rejection-related interpersonal stressors. On the other hand, different types of interpersonal stressors may impart similar risk for suicidal ideation. Our results extend prior studies using timeline followback methods that have identified interpersonal stressors as a proximal warning sign for suicide attempts to suggest that targeted rejection is a particularly important type of interpersonal stress that increases proximal risk for suicidal behavior (see Bagge et al., 2013; Conner et al., 2012).

Together, our findings suggest that both targeted rejection and broad-based interpersonal stressors are associated with the same-day risk for suicidal ideation, potentially on even shorter timescales, whereas targeted rejection increases same-day risk for suicidal behaviors that is sustained over the next day. These findings extend prior empirical research and suicide theories that primarily emphasize interpersonal stressors in the pathway to suicidal desire, to suggest that targeted rejection may actually be an important prospective near-term risk factor for engaging in suicidal behavior as well. In line with Social Safety Theory (Slavich et al., 2023), targeted rejection may uniquely impact risk for suicidal behaviors because of its strong impact on social connection (i.e. the loss of a social tie or social demotion) and other key characteristics (e.g. intent, isolated impact). Consistent with an Integrated Model of Social Exclusion and Suicide (Brown, 2019), we postulate that targeted rejection, due to its potency, may increase risk for suicidal behaviors by exerting its effects on not only suicidal thinking, which may occur over shorter periods of time, but also on suicide capability factors (e.g. increased negative urgency or impulsivity, impaired decision-making) that facilitate the transition from suicidal ideation to suicidal behaviors. Further research is warranted to better articulate the role of targeted rejection in the pathway to suicidal behavior.

It is also noteworthy that all effects of stressor severity were observed on the within-person level, with no significant effects of individual differences in overall levels of stressor severity on mean propensity for suicidal ideation or behavior emerging on the between-persons level of analysis. This might suggest that the association between stressor exposure and suicide risk is more dynamic, rather than static or trait-like, which is consistent with suicide theories that emphasize proximal links between life stress and suicide risk (Klonsky, 2018). Nonetheless, the lack of effects at the between-person level should be interpreted with caution due to limited power to detect associations at this level of analysis (given that power is not enhanced with repeated observations, as it is on the within-persons level, where we have 64,731 observations). For this reason, the small effect sizes we observed on the within-person level, while statistically significant, may not be clinically meaningful given that the high statistical power can reveal even very small effects. More intensive longitudinal research on objective stressor exposure as a near-term risk factor for suicidal outcomes is needed to better understand the impact of fluctuations in stressor exposure on short-term suicide risk states versus longer-term distal risk.

### Strengths and limitations

The present study has several strengths that address notable gaps and weaknesses in prior research. These include our use of a gold-standard, investigator-based system for objectively assessing life stressor exposure; assessing theoretically important types of stressors; examining stress and suicide outcomes on a daily timescale; and investigating suicidal ideation and behavior as separate outcomes. Moreover, this is the first study to demonstrate that objectively rated stressor severity is proximally associated with increased risk for suicidal ideation and behavior on a daily timescale, even after controlling for prior-day suicidal ideation and behavior. The effects also remained significant even when we added potent suicide risk factors (e.g. non-suicidal self-injury, alcohol use) as time-varying covariates. The data thus provide strong evidence for the potency of stressful life events, especially targeted rejection events, as proximal risk factors for suicide that cannot be explained by other factors such as mood-dependent recall biases that may cause individuals at greater risk for suicidal ideation and behavior to remember or perceive specific life events as more stressful than those at lower risk. Indeed, the present study’s combination of objective threat and timeline followback interviews provides an innovative approach for future investigations of objectively rated stressors as proximal risk factors for suicide and other health-related outcomes.

One significant limitation of this study is that the interpretation of results for suicidal ideation rests on the assumption that participants were able to accurately recall suicidal ideation at the daily level using the timeline followback interview at 4-month intervals. The assessment of nonbehavioral outcomes (e.g. thoughts) that may occur at high frequency and fluctuate rapidly over time is best measured as close in time to the outcome as possible. There is also the possibility that participants may have been more likely to recall suicidal ideation that occurred in close proximity to stressful life events that serve as anchors in the recall of events, which may introduce bias. Alternatively, retrospective recall may have led to underreporting of very brief suicidal ideation. It is thus important that future studies examine the reliability and validity of timeline followback methods for assessing suicidal ideation and behavior retrospectively at longer time intervals from the target behavior and evaluate potential bias in combining this assessment with the recall of major life events.

Another limitation is the low base rate of suicidal behavior in the present study, which necessitated the combination of suicidal behavior types for analysis, leading to uncertainty regarding which specific suicidal behaviors are driving results. It will thus be important for future work to examine whether the effects of stressor exposure differ for preparatory behaviors (e.g. collecting pills, writing a suicide note) and different types of suicide attempts. We also note that interrater reliability for targeted rejection was modest, which raises some uncertainty regarding the robustness of our findings. Our reliability ratings for targeted rejection were based on more limited information (i.e. written narratives) than the original team ratings (i.e. live discussion). Future studies might improve reliability by having independent groups of raters code the exact same information to improve the reliability of the measures. Given this limitation, replication of these findings is critical to drawing firm conclusions about clinical implications. We also note that given the mostly female sample, while representative of outpatient treatment–seeking populations at risk for suicide, our findings may not generalize to men. It will be important for future studies to examine how these effects generalize across various sample characteristics, including sex, age, and level of suicide risk. A final limitation is that we did not assess chronic stressors, which are strongly related to suicide outcomes (Stewart et al., 2024), or biological processes that may link stressor exposure and suicidal outcomes.

### Conclusion

Notwithstanding these limitations, this is the first study we know of to demonstrate that objectively rated interpersonal stressors are proximal risk factors for both suicidal ideation and behavior in young adults and, additionally, that targeted rejection may be especially strongly related to suicidal behavior. Consequently, clinicians assessing acute suicidal risk should evaluate if patients have experienced a recent interpersonal life event or, even more critically, a recent targeted rejection life event, such as being broken up with or having been fired. More broadly, interventions aimed at preventing suicide may benefit from stabilizing social relationships to reduce social rejection and foster social safety, and helping patients better regulate their emotions when acute interpersonal stressors occur (e.g. using real-time adaptive interventions). Finally, the present data can help theories of suicide risk to (a) further refine the timescale on which interpersonal stressors increase suicide risk and (b) incorporate targeted rejection as a potential near-term risk factor for suicidal behavior. Looking forward, additional research is needed to examine the cognitive, affective, and biological mechanisms by which interpersonal stressors, and especially targeted rejection stressors, increase risk for suicidal ideation and behavior.

## Supplementary Material

Supplemental material

**Supplementary material.** The supplementary material for this article can be found at http://doi.org/10.1017/S0033291725101414.

## Figures and Tables

**Table 1.** Demographic and clinical diagnostic information at study baseline (*N* = 143)

	*n* (%)
Sex assigned at birth	
Male	24 (16.8)
Female	119 (83.2)
Gender	
Male	24 (16.8)
Female	106 (74.1)
Gender diverse	13 (9.1)
Ethnicity	
Non-Hispanic/LatinX	130 (90.9)
Hispanic/LatinX	13 (9.1)
Race	
Asian	12 (8.4)
Black or African American	23 (16.1)
White	97 (67.8)
More than one race	10 (7.0)
Prefer not to respond	1 (0.7)
Sexual Orientation	
Heterosexual/straight	67 (46.9)
Gay/lesbian/homosexual	14 (9.8)
Bisexual	39 (27.3)
Other	17 (11.9)
Not sure	4 (2.8)
Declined	1 (0.7)
Education	
Less than high school or GED	1 (0.7)
High school or GED	11 (7.7)
Some college	49 (34.3)
Technical/trade school certificate	4 (2.8)
College degree or higher	78 (54.5)
Employment	
Not employed	40 (27.9)
Part-time	47 (32.9)
Full-time	56 (39.2)
Annual Income: *M (SD)*	$73,084 ($76,906)
Public Assistance (e.g. Medicaid, WIC) in last year	48 (33.6)
C-SSRS Suicidal ideation (SI) highest level (Past 4 Months)	
Wish to be dead	24 (16.8)
Nonspecific active SI	24 (16.8)
Active SI with methods (no plan or intent)	43 (30.1)
Active SI with some intent (no plan)	44 (30.8)
Active SI with specific plan and intent	8 (5.6)
C-SSRS suicidal behavior (past 4 months)	
Actual attempt	7 (4.9)
Interrupted attempt	4 (2.9)
Aborted attempt	8 (5.6)
Preparatory behavior	15 (10.5)
C-SSRS suicidal behavior (lifetime)	
Actual attempt	52 (36.4)
Interrupted attempt	17 (11.9)
Aborted attempt	42 (29.4)
Preparatory behavior	57 (39.9)
SCID-5-RV current diagnosis	
Mood disorder	92 (64.3)
Anxiety disorder	94 (65.7)
Obsessive-compulsive related disorder	18 (12.6)
Trauma and stress-related disorder	34 (23.8)
Substance use disorder	41 (28.7)
Eating disorder	8 (5.6)
SCID-5-RV lifetime diagnosis	
Mood disorder	142 (99.3)
Anxiety disorder	113 (79.0)
Obsessive-compulsive related disorder	30 (21.0)
Trauma and stress-related disorder	67 (46.9)
Substance use disorder	77 (53.8)
Eating disorder	44 (30.8)
SIDP-IV current diagnoses	
Borderline personality disorder	21 (14.7)
Antisocial personality disorder	5 (3.5)
Avoidant personality disorder	35 (24.5)

*Note: N* = 143. C-SSRS, Columbia Suicide Severity Rating Scale (Posner et al., 2011); SCID-5-RV, Structured Clinical Interview for DSM-5 (First et al., 2015); SIDP-IV, Structured Clinical Interview for DSM-IV Personality Disorders (Pfohl et al., 1997), the criteria for which are unchanged in DSM-5. Percentages may not sum to 100% due to missing data and overlapping categories (e.g. co-occurring diagnoses).

**Table 2.** Rates of endorsement of daily level constructs (*N* = 143 persons; *N* = 64,731 days) retrospectively reported in timeline followback (TLFB) interviews

	*N* (%) subjects	*N* (%) observations (days)
Suicidal Ideation	143 (100%)	13,899 (21.5%)
Suicidal behavior	45 (31.5%)	104 (0.2%)
Preparatory behavior	23 (16.1%)	51 (0.1%)
Aborted attempt	18 (12.6%)	27 (<0.1%)
Interrupted attempt	6 (4.2%)	10 (<0.1%)
Actual attempt	13 (9.1%)	17 (<0.1%)
Non-suicidal self-injury	57 (39.9%)	886 (1.4%)
Alcohol use (1 or more standard drinks)	127 (88.8%)	7,374 (11.4%)
Psychoactive drug use	113 (79%)	20,592 (31.8%)

**Table 3.** Descriptive statistics and bivariate correlations at the between-person level (*N* = 143)

	1	2	3	4	5	6	7	8	9	10	11	12	13
1. Number of days of data	–												
2. Public assistance due to low income	−.01	–											
3. Female gender identification	−.06	.01	–										
4. Gender diverse identification	.03	.03	−.54**	–									
5. Suicidal ideation	−.26**	.04	−.07	.01	–								
6. Suicidal behavior	−.18*	.19*	−.22**	.19*	.29**	–							
7. Non-suicidal self-injury	−.13	.12	.03	.01	.08	.10	–						
8. Drug use	.06	.15	.00	.19*	.13	.05	.29**	–					
9. Number of standard alcohol drinks	−.08	−.10	−.17*	.02	.00	−.01	−.04	.01	–				
10. Non-interpersonal stressors	−.10	−.03	−.12	.07	.09	.07	.01	−.07	.47**	–			
11. Non-rejection interpersonal stressors	−.22**	.04	.09	−.06	.12	.17*	.01	−.11	.05	.20*	–		
12. Social rejection stressors without targeted rejection	−.01	−.03	−.17*	.33**	.04	.23**	.03	.01	.04	.06	.01	–	
13. Targeted rejection stressors	.01	.16	−.13	.17*	.06	.11	.02	.15	−.06	−.06	−.02	.24**	–
%/*M*	452.66	33.6%	74.1%	9.1%	0.23	0.002	0.02	0.31	0.32	0.06	0.06	0.01	0.01
*SD*	112.75				0.24	0.004	0.05	0.37	0.64	0.10	0.09	0.04	0.01

*Note:* N days of data, Number of days of timeline followback data. Repeated daily measures were aggregated by person by calculating the mean across assessments.

* *p* < .05

** *p* < .01

**Table 4.** Stressor severity as predictors of same-day and next-day suicidal ideation and behavior

Same-day model	Suicidal ideation			Suicidal behavior		
	Est (*SD*)	95% CI (lower, upper)	*OR*	Est (*SD*)	95% CI (lower, upper)	*OR*
Suicidal ideation on suicidal ideation_lag_/suicidal behavior on suicidal behavior_lag_	0.95 (0.003)**	0.94, 0.95	4.62	0.69 (0.04)**	0.60, 0.77	3.04
Non-interpersonal stressors	0.04 (0.02)*	0.004, 0.07	1.07	0.14 (0.04)**	0.06, 0.22	1.25
Non-rejection interpersonal stressors	0.09 (0.02)**	0.06, 0.13	1.16	0.09 (0.05)	−0.02, 0.19	1.16
Social rejection stressors without targeted rejection	0.11 (0.04)**	0.04, 0.18	1.19	0.14 (0.08)	−0.04, 0.29	1.25
Targeted rejection stressors	0.17 (0.06)**	0.05, 0.29	1.31	0.26 (0.09)**	0.08, 0.42	1.52
Next-day model						
Suicidal ideation on suicidal ideation_lag_/suicidal behavior on suicidal behavior_lag_	0.95 (0.003)**	0.94, 0.96	4.62	0.68 (0.05)**	0.56, 0.74	2.99
Non-interpersonal stressors_lag_	0.004 (0.02)	−0.03, 0.04	1.01	0.08 (0.05)	−0.02, 0.16	1.14
Non-rejection interpersonal stressors_lag_	0.02 (0.02)	−0.01, 0.06	1.03	−0.01 (0.07)	−0.16, 0.10	0.98
Social rejection stressors without targeted rejection_lag_	−0.03 (0.04)	−0.10, 0.04	0.95	0.07 (0.09)	−0.13, 0.22	1.12
Targeted rejection stressors_lag_	0.09 (0.06)	−0.03, 0.20	1.16	0.25 (0.09)**	0.08, 0.41	1.50

*Note:* Est (*SD*), Unstandardized within-person parameter estimates (probit) and their posterior standard deviations; *OR*, odds ratio; CI, credibility interval.

* *p* < .05

** *p* < .01.
