# Supplementary material for: Interpersonal and targeted rejection life stressors are proximal risk factors for suicidal ideation and behavior
Source: Psychol Med. 2025 Sep 12;55:e271. doi: 10.1017/S0033291725101414 (PMC12517110; doi:10.1017/S0033291725101414)
Supplement: Scott et al. supplementary material [file S0033291725101414sup001.docx]

**Supplemental Materials**

**for**

**Interpersonal and targeted rejection life stressors are proximal risk factors for suicidal ideation and behavior**

**Supplemental Table 1**

*Categorization of Episodic Life Events (N = 1,989)*

|  | Frequency | Percent |
| --- | --- | --- |
| Event duration (days) |  |  |
| 1 | 1644 | 82.7 |
| 2-10 | 274 | 13.7 |
| 11-20 | 47 | 2.4 |
| 21-30 | 12 | 0.6 |
| 31+ | 12 | 0.6 |
| Consensus Objective Stress |  |  |
| 1.5 | 906 | 45.6 |
| 2.0 (Mild) | 617 | 31 |
| 2.5 | 268 | 13.5 |
| 3.0 (Moderate) | 105 | 5.3 |
| 3.5 | 44 | 2.2 |
| 4.0 (Marked) | 35 | 1.8 |
| 4.5 | 8 | 0.4 |
| 5.0 (Severe) | 6 | 0.3 |
| Interpersonal | 1197 | 60.2 |
| Focus |  |  |
| Subject | 1154 | 58.0 |
| Joint (subject and other) | 688 | 34.6 |
| Other social network | 147 | 7.4 |
| Life Domain (more than 1 could apply) |  |  |
| Housing | 120 | 6.0 |
| Education | 120 | 6.0 |
| Work | 277 | 13.9 |
| Treatment/Health | 452 | 22.7 |
| Marital/Partner/Romantic | 406 | 20.4 |
| Reproduction | 27 | 1.4 |
| Financial | 191 | 9.6 |
| Legal/Crime | 99 | 5.0 |
| Other Relationships | 525 | 26.4 |
| Death | 102 | 5.1 |
| Life Threatening | 20 | 1.0 |
| Possessions | 113 | 5.7 |
| Fear Evoking | 37 | 1.9 |
| Assault | 43 | 2.2 |
| Physically Painful | 183 | 9.2 |
| Other Life Domain | 24 | 1.2 |

**Supplemental Table 2**

*Characterization of interpersonal life events (N = 1,197)*

|  | Frequency | Percent |
| --- | --- | --- |
| With whom (more than 1 could apply) |  |  |
| Friend/Peer | 262 | 21.9 |
| Romantic Partner | 425 | 35.6 |
| Family | 367 | 30.7 |
| Other | 231 | 19.3 |
| Social Rejection |  |  |
| Intent to Reject | 234 | 19.6 |
| Isolated Impact | 227 | 19.0 |
| Social Demotion | 101 | 8.4 |
| Targeted Rejection | 99 | 8.3 |

**Supplemental Table 3**

*Examples of stressful life events by interpersonal category*

| Event type | Description of event |
| --- | --- |
| Non-interpersonal | Started new job |
|  | Moving into own apartment for the first time |
|  | Exams/midterms |
|  | Surgery for tumor removal |
|  | Put in offer on first house |
| Interpersonal without social rejection | Job interview |
|  | Breakup with romantic partner (mutual) |
|  | Attended a funeral |
|  | Family conflict/argument |
|  | Physically assaulted |
|  | Death of a close loved one |
| Social rejection without targeted rejection | Withdrawn from consideration for position after job interview (no social demotion) |
|  | Received insulting email from family member, no severing of relationship (no social demotion) |
|  | Grew apart from friend (no intent to reject) |
|  | Someone posted negative accusations about them online, but did not lead to loss of relationships (no social demotion) |
|  | Received warning for poor performance at work and put on performance improvement plan (no social demotion)  Laid off from job along with many other employees due to company-wide cutbacks (no isolated impact) |
| Targeted rejection | Romantic partner broke up with them |
|  | Fired from job |
|  | Friend blocked them on phone/social media |
|  | Coworker ended friendship |
|  | Negative social media post about them went viral, lost friends as result |
